# Supplementary material for: Genomic prediction of celiac disease targeting HLA-positive individuals
Source: Genome Med. 2015 Jul 16;7(1):72. doi: 10.1186/s13073-015-0196-5 (PMC4523954; doi:10.1186/s13073-015-0196-5)

Genomic prediction of celiac disease targeting HLA-positive individuals

Gad Abraham, Alexia Rohmer, Jason A. Tye-Din, Michael Inouye

Supplementary Results

Assessment of potential reasons for the reduction in AUC on the North American dataset

We sought to investigate the source for the small reduction in AUC observed in the North American dataset relative to the European data. First, we employed the same methods used to develop the GRS14 (L1-penalized linear support vector machines) to generate a CD risk score within the North American dataset; specifically, we used 10-fold cross-validation within the North American dataset to estimate the predictive power (area under receiver-operating-characteristic curve, AUC) of the penalized models, as a function of the number of SNPs assigned a non-zero weight in the model (**Supplementary Figure 3**). The best average AUC of 0.823 was achieved with models including ~40 SNPs with non-zero weights. Beyond that, increasing the number of SNPs with non-zero weight in the model reduced the cross-validated AUC, indicating overfitting. As further verification that the difference in AUC was not driven by our choice of model, we employed MultiBLUP, a non-sparse genome-wide modeling method based on linear mixed models, on the European GWA data (n=11,912), and tested its predictions on the North American cohort. MultiBLUP achieved AUC = 0.831 (95% CI 0.808—0.853), equal to the AUC of the GRS14. Next, we examined the distribution of the GRS14 scores, stratified by CD diagnosis method (controls, biopsy & serology, biopsy, serology, and unknown), to examine whether there were substantial differences in predicted risk between diagnosis methods that could indicate potential misclassification of case/control due to variability in diagnosis method. We did not observe substantial differences in the median risk between diagnosis methods apart from the expected difference between cases (regardless of diagnosis method) and controls (**Supplementary Figure 4**). Finally, we computed the fixation index F_{st} for the 224 SNPs in the risk score between the North American and European data (mean $F_{st}=3\times10^{-3}$ based on 213 SNPs with valid estimates), confirming the earlier PCA results showing negligible allele frequency differences between the training and validation datasets.

GRS-DQ2.5 based on a combined Immunochip and GWA training set

We also explored whether using a DQ2.5-specific GRS trained on the combined European GWA and Immunochip datasets, employing the SNPs common to the two platforms and other SNP2HLA markers (10,284 samples and 24,555 SNPs+markers), would lead to increased predictive power over using just the European GWA data. While training SparSNP on this combined SNP+marker dataset led to increases in cross-validated AUC (0.748 for an L2 penalty of 0.01; **Supplementary Figure 6**), this model subsequently achieved lower AUC in external validation on the North American dataset (AUC = 0.707, 95% CI 0.663—0.750).

Supplementary Figure 1: The first two principal components (PCs) of an LD-thinned dataset combining the European GWA datasets (Finland, Italy, Netherlands, and UK) and the North American dataset (post QC).

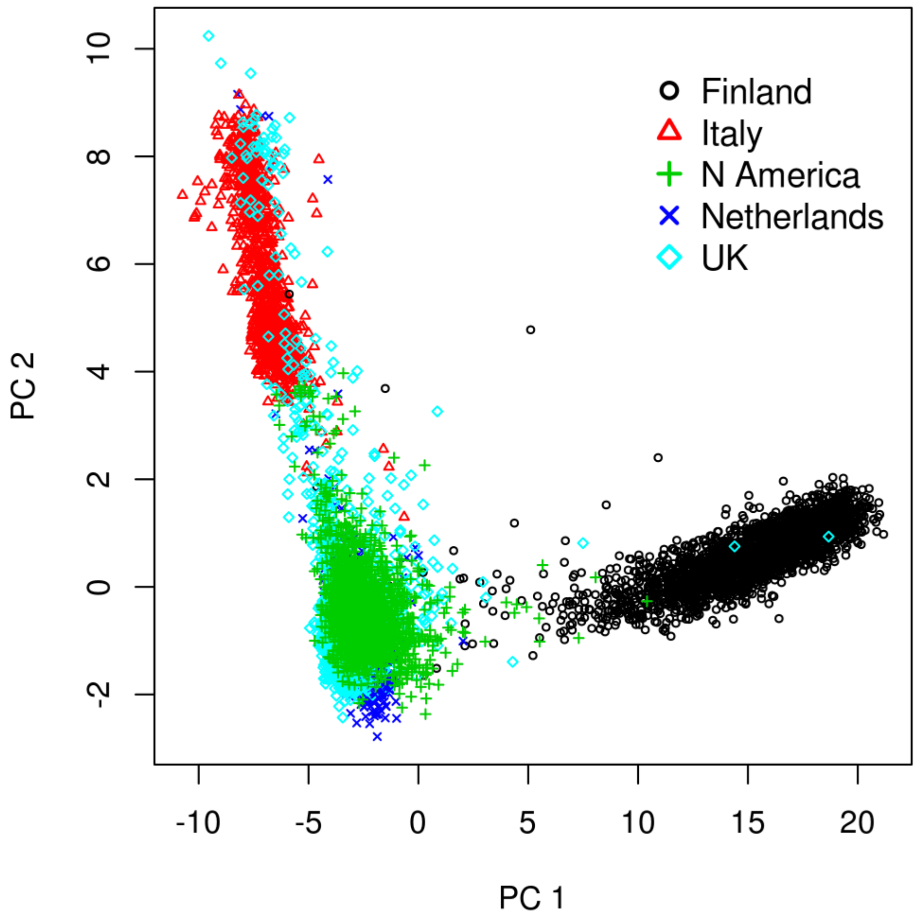

Supplementary Figure 2: ROC curves for classifying all CD cases and controls using different predictors in the North American dataset.

GRS14: the published GRS (trained on the UK2 dataset); *GRS IMM+UK2*: a GRS trained on the Immunochip + UK SNP data; *GRS-imputed GWA:* a GRS trained on all European GWA datasets (UK, Dutch, Finnish, Italian), consisting of SNPs and SNP2HLA imputed markers; *GRS-imputed IMM+GWA*: a GRS trained on the European GWA + Immunochip datasets, consisting of SNPs and SNP2HLA imputed markers; *HLA haplotype risk:* a 3-level risk score based on the imputed HLA haplotype status; *MultiBLUP GWA*: a MultiBLUP model trained on the European GWA data.

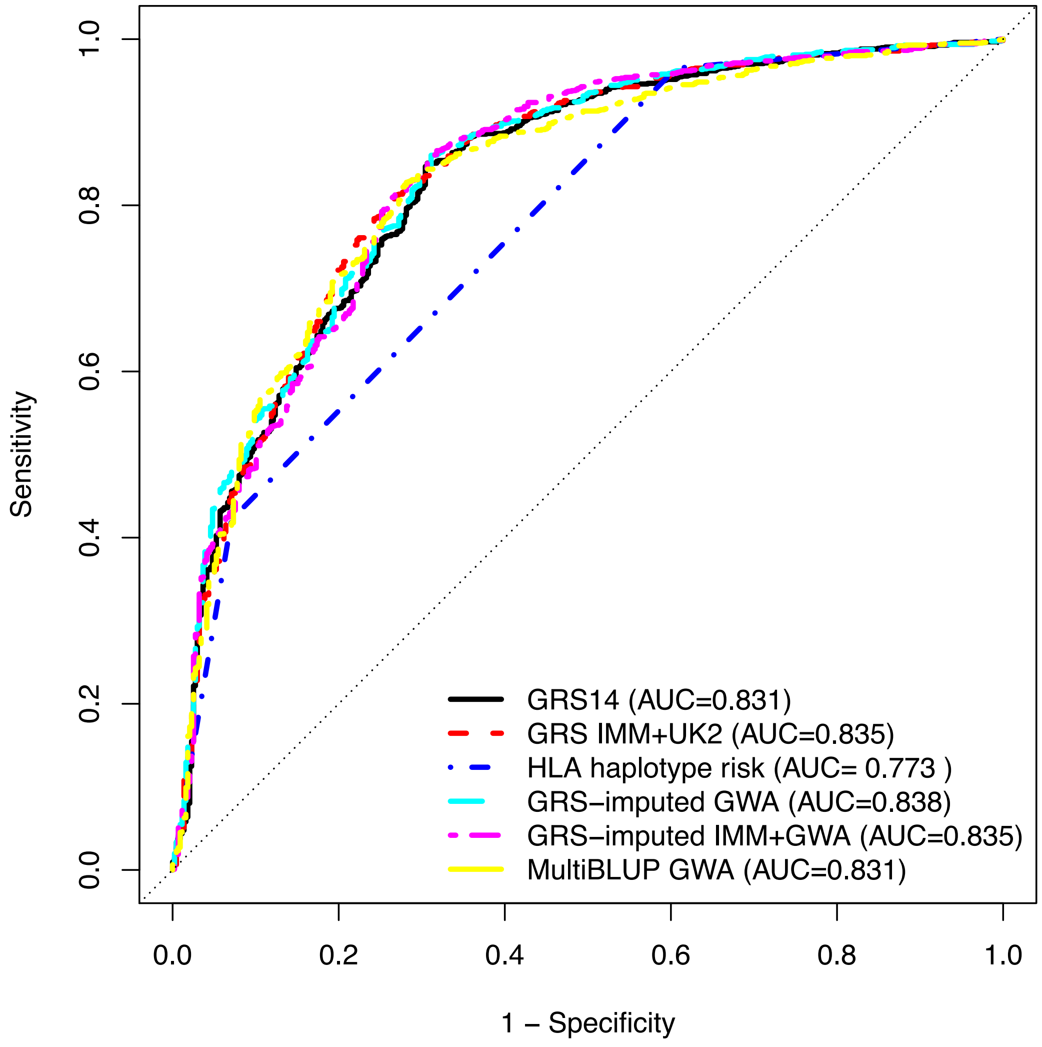

Supplementary Figure 3: 10×10 cross-validated AUC (LOESS-smoothed) for the GRS developed within the North American dataset, as a function of the number of SNPs assigned a non-zero weight in the model. The maximum AUC was 0.823 at 40 SNPs with non-zero weight.

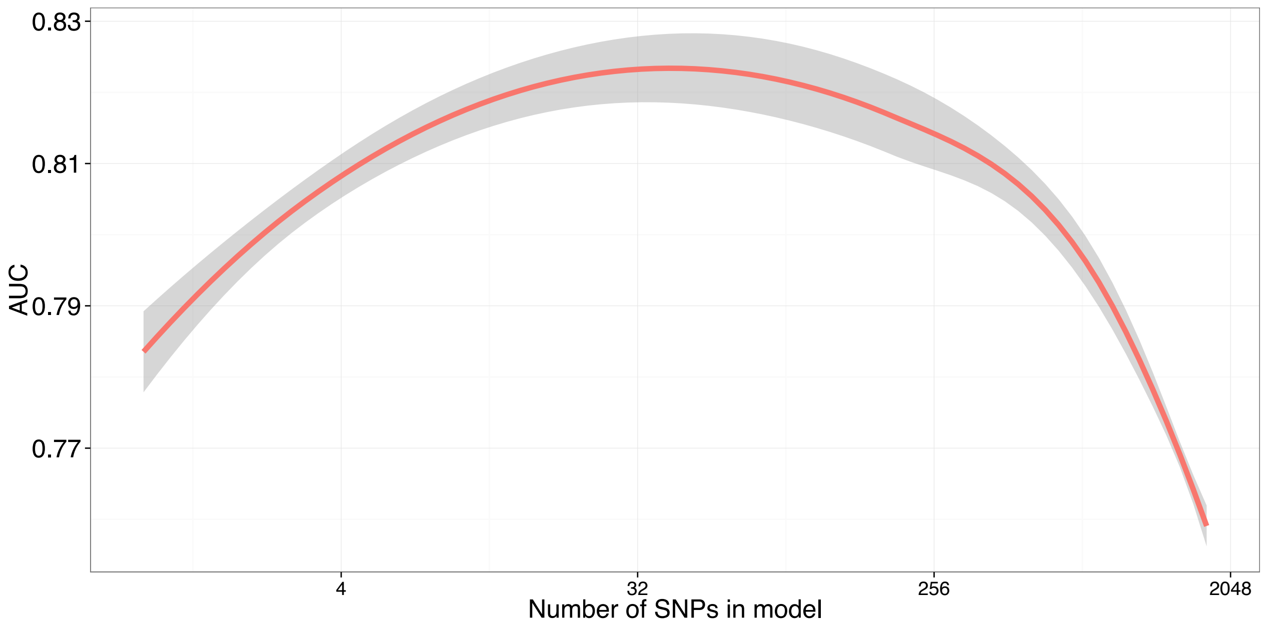

Supplementary Figure 4: Boxplots of the genomic risk scores (GRS14) within each diagnosis method for all individuals in the North American cohort (n=1696).

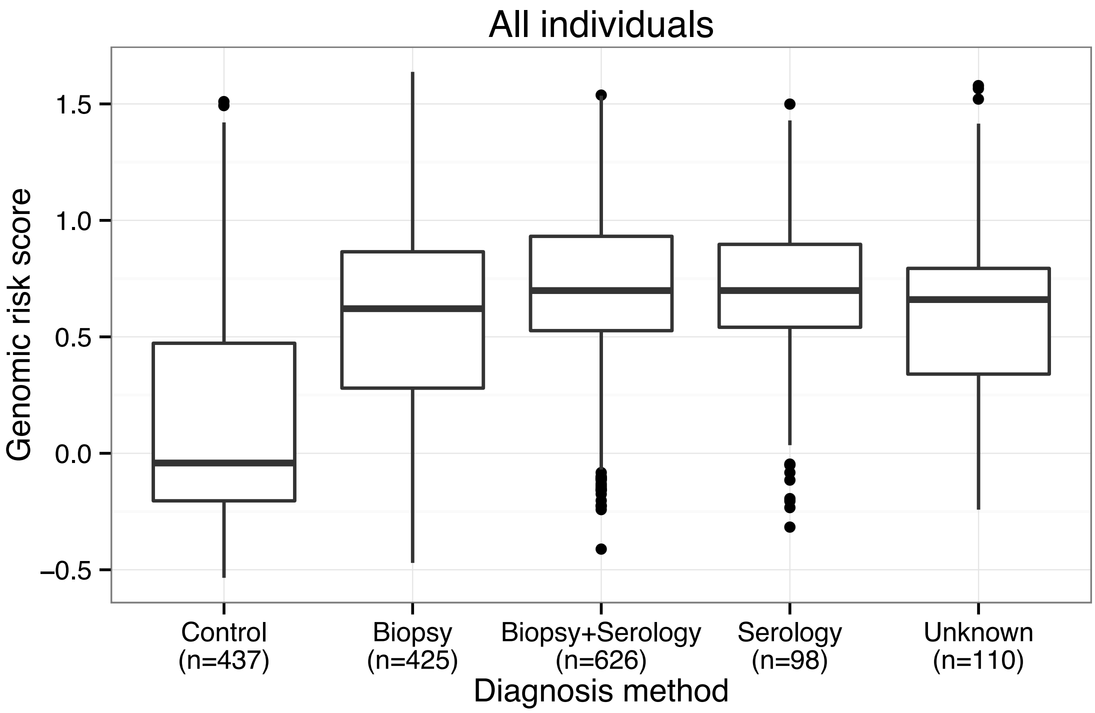

Supplementary Figure 5: 10×10 cross-validated AUC (LOESS-smoothed) for 10 candidate GRS developed on the DQ-2.5+ individuals from the European GWA SNP datasets (n=5552), each using a different L2 penalty.

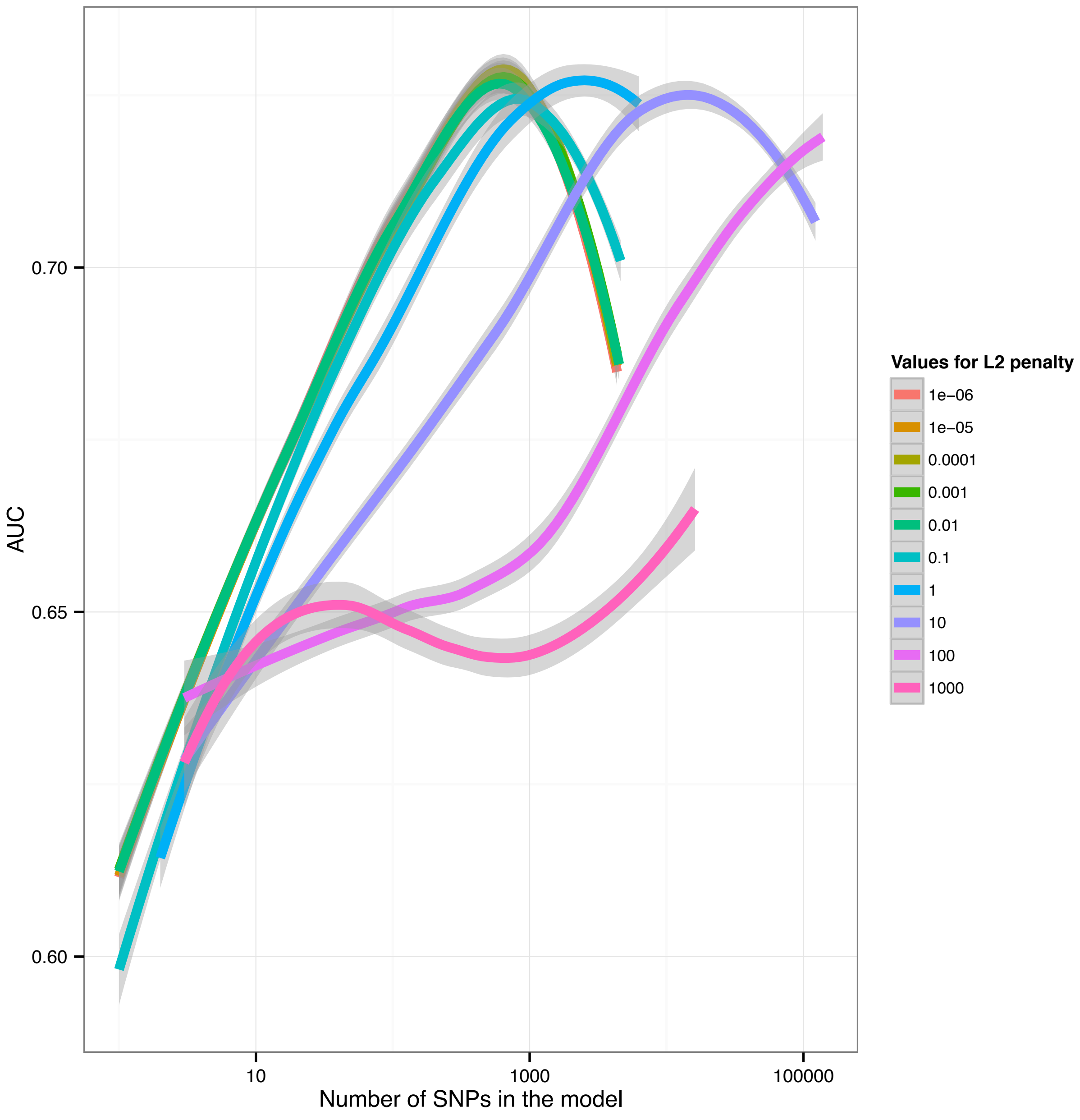

Supplementary Figure 6: 10×10 cross-validated AUC (LOESS-smoothed) for the novel GRS-DQ2.5 model trained on the combined DQ2.5+ subsets of the European GWA data and the Immunochip data (n=10,284), as a function of the number of SNPs assigned a non-zero weight in the model. For the SNP+marker model, maximum AUC of 0.742 was achieved at 583 SNPs/markers with non-zero weight using an L2 penalty of 0.01.

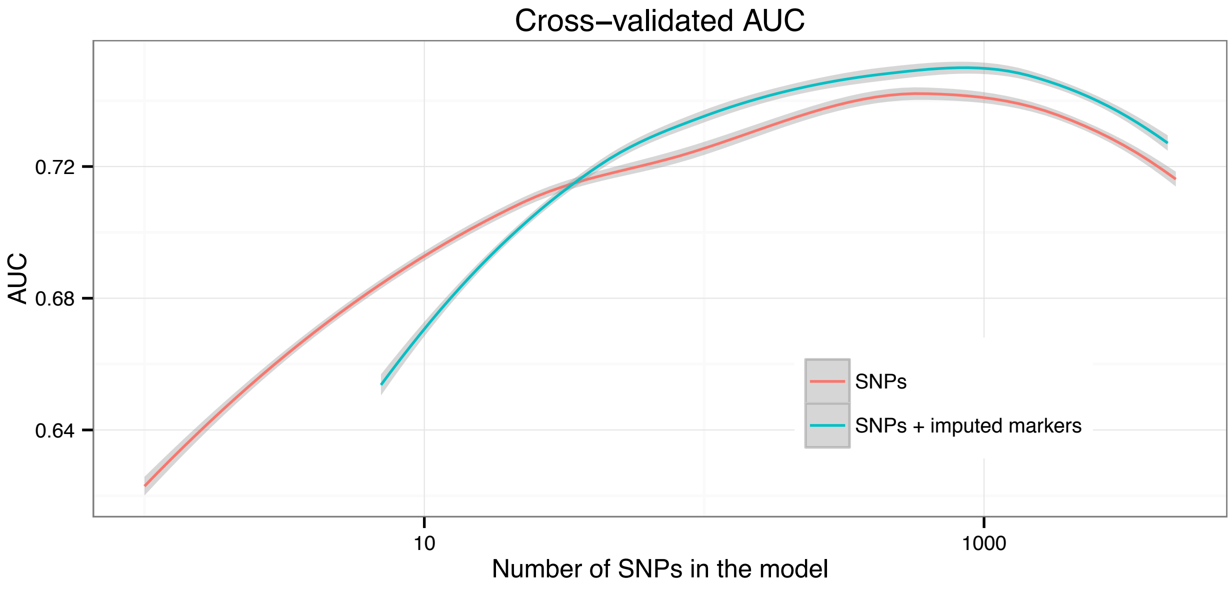

Supplementary Figure 7: External validation results on the DQ2.5+ individuals in the North American dataset, focusing on sensitivity ≥90%.
(**a**) ROC curves for case/control prediction and (**b**) Non-CD implicated per CD correctly implicated, ((1 – PPV) / PPV, equivalent to 1 / [post-test-odds of disease]) versus sensitivity, for models developed on the European data and tested on the DQ2.5+ subset of the North American cohort. The DQ2.5 zygosity is the number of DQ2.5 alleles for each individual (heterozygous=1, homozygous=2). We assumed a CD prevalence of 10% in the DQ2.5+, corresponding to a baseline implication ratio of 9:1, that is, all DQ2.5+ implicated as having CD at 100% sensitivity. Note that the estimate of HLA haplotype risk for (b) fall below the sensitivity of 90% and are not shown.

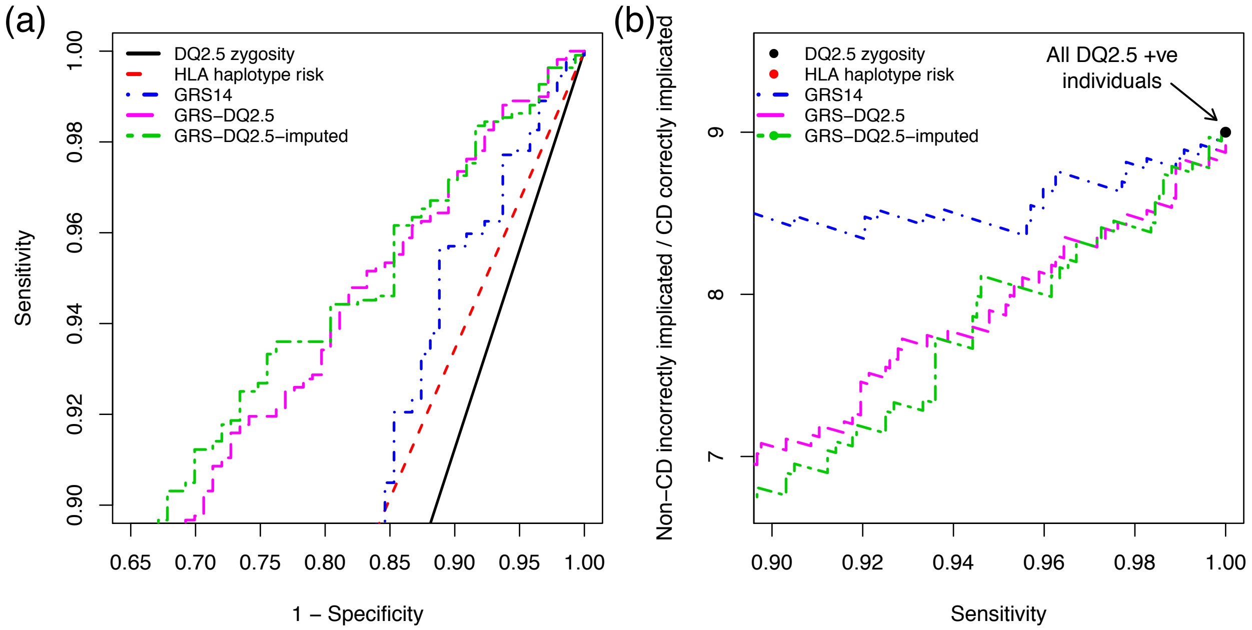

Supplement: Additional file 1: — Supplementary results and figures. (PDF 437 kb) [file 13073_2015_196_MOESM1_ESM.pdf]
